# Intravenous administration of mesenchymal stem cells prevents angiotensin II-induced aortic aneurysm formation in apolipoprotein E-deficient mouse

**DOI:** 10.1186/1479-5876-11-175

**Published:** 2013-07-22

**Authors:** Xian-ming Fu, Aika Yamawaki-Ogata, Hideki Oshima, Yuichi Ueda, Akihiko Usui, Yuji Narita

**Affiliations:** 1 Department of Cardiothoracic Surgery, Nagoya University Graduate School of Medicine, 65 Tsurumai-cho, Showa-ku, Nagoya Aichi 466-8550, Japan

**Keywords:** Mesenchymal stem cells, Aortic aneurysm, Cell transplantation, Chronic inflammation, Matrix metalloproteinase

## Abstract

**Background:**

Mesenchymal stem cells (MSCs) are known to be capable of suppressing inflammatory responses. We previously reported that intra-abdominal implantation of bone marrow-derived MSCs (BM-MSCs) sheet by laparotomy attenuated angiotensin II (AngII)-induced aortic aneurysm (AA) growth in apolipoprotein E-deficient (apoE^−/−^) mice through anti-inflammation effects. However, cell delivery by laparotomy is invasive; we here demonstrated the effects of multiple intravenous administrations of BM-MSCs on AngII-induced AA formation.

**Methods:**

BM-MSCs were isolated from femurs and tibiae of male apoE^−/−^ mice. Experimental AA was induced by AngII infusion for 28 days in apoE^−/−^ mice. Mice received weekly intravenous administration of BM-MSCs (n=12) or saline (n=10). After 4 weeks, AA formation incidence, aortic diameter, macrophage accumulation, matrix metalloproteinase (MMP)’ activity, elastin content, and cytokines were evaluated.

**Results:**

AngII induced AA formation in 100% of the mice in the saline group and 50% in the BM-MSCs treatment group (*P* < 0.05). A significant decrease of aortic diameter was observed in the BM-MSCs treatment group at ascending and infrarenal levels, which was associated with decreased macrophage infiltration and suppressed activities of MMP-2 and MMP-9 in aortic tissues, as well as a preservation of elastin content of aortic tissues. In addition, interleukin (IL)-1β, IL-6, and monocyte chemotactic protein-1 significantly decreased while insulin-like growth factor-1 and tissue inhibitor of metalloproteinases-2 increased in the aortic tissues of BM-MSCs treatment group.

**Conclusions:**

Multiple intravenous administrations of BM-MSCs attenuated the development of AngII-induced AA in apoE^−/−^ mice and may become a promising alternative therapeutic strategy for AA progression.

## Background

Aortic aneurysm (AA) is a common life-threatening disease and its prevalence is increasing with the ageing society [1]. For large AAs, which have a high risk of rupture, surgical treatment by open or endovascular repair is generally recommended. However, the great majority of AA is small and below the threshold for immediate surgical repair. Early elective surgical repair of small AA does not provide long term survival advantages [2,3]. On the other hand, there are few therapeutic strategies for surgically unsuitable patients who show high risk for surgery. Therefore, the development of a non-surgical therapeutic approach for treating AA is needed. With advances in vascular biology and understanding of the mechanism of AA formation at the cellular and molecular levels, several pharmacological agents, including statins, β-blockers, ACE-inhibitors, anti-inflammatory agents, and antibiotics, have been reported to limit AA progression in experimental models and some agents have undergone clinical trials [4,5]. However, they showed modest benefits in large clinical trials [5,6], and no medicine is currently approved for the prevention of AA progression.

The pathogenesis of AA is characterized by chronic inflammation in the aortic wall with accumulation of macrophages and degradation of extracellular matrix with increased matrix metalloproteinases (MMPs). Infiltrated inflammatory cells such as macrophages, and lymphocytes cause activation of matrix metalloproteinases, particularly MMP-2 and MMP-9, resulting in the degradation of both collagen and its associated collagenous matrix, along with elastin fragmentation and smooth muscle cell (SMCs) apoptosis, which contribute prominently to AA development [7-9]. The positive correlation between inflammatory infiltrates and aneurysmal enlargement [10], as well as regression of established AA by limiting proinflammatory signaling in mice [11], suggest the important role of inflammation in AA pathogenesis.

Mesenchymal stem cells (MSCs) are adult somatic cells that reside in the stroma of solid organs, and have been demonstrated to differentiate into a variety of cell types including: osteoblasts, chondrocytes, and adipocytes [12]. Recently, MSCs have been shown to have anti-inflammation effects, and have been applied for treating immune/inflammation associated disorders such as sepsis [13], graft versus host disease [14], and experimental colitis [15]. We previously demonstrated that intra-abdominal implantation of BM-MSCs sheet by laparotomy could attenuate the development of angiotensin II (AngII) -induced AA in apolipoprotein E-deficient (apoE^−/−^) mice by anti-inflammation [16]. However, this approach requires surgery to deliver BM-MSCs, and may be invasive to patients. Intravenous delivery of BM-MSCs offers an attractive approach, because it is simple, less invasive and is a repeatable administration of a large numbers of cells. The property of BM-MSCs mobilizing or homing into sites of tissue damage or inflammation raises the possibility of intravenous administration of BM-MSCs for treatment of AA [17].

Therefore, in this study, we investigated the effects of intravenous administration of BM-MSCs on AngII- induced AA formation in apoE^−/−^ mice.

## Methods

### Animal

ApoE^−/−^ mice (genetic background C57BL/6) were obtained from the Jackson Laboratory (Sacramento, California) and maintained on a regular chow diet under standard conditions. All animal experiments were performed in accordance with the Guide for the Care and Use of Laboratory Animals published by the US National Institutes of Health (NIH publication No. 85–23, revised 1996), and approved by the Animal Care and Use Committee of Nagoya University (protocol No. 24061).

### Isolation, culture, and differentiation of bone marrow-derived mesenchymal stem cells

BM-MSCs were isolated from femurs and tibiae of male apoE^−/−^ mice (6 to 8 weeks old) and expanded as described [18]. The mesenchymal population was isolated on the basis of its ability to adhere to the culture plate. At 90% confluence, the cells were trypsinized (0.05% Trypsin containing 0.53 mM EDTA-4Na, Invitrogen) and were passaged to 175-cm^2^ flasks. Expansion culture continued up to passages 7–10 were used in all experiments. Cultured BM-MSCs were incubated with the following antibodies, conjugated with phycoerythrin (PE), allophycocyanin (APC) or peridinin chlorophyll protein-Cy5.5 (PerCP-Cy5.5) (eBioscience): CD11b, CD31, CD34, CD44, CD45, CD73, CD106, CD117, and Sca-1. Labeled cells were assayed by flow cytometer (FACS Canto II, Becton Dickinson) and analyzed with BD FACSDiva Software and FlowJo (Tree Star Inc.). The capacity to differentiate into osteogenic, adipogenic, and chondrogenic lineages by addition of inductive media was confirmed with chemical staining as performed previously (Additional file 1) [18].

### AA model and MSCs transplantation

Ang II induced aortic aneurysm model in apoE^−/−^ mice were used in this study. Male apoE^−/−^ mice (24 to 28 weeks old) were infused with 1000 ng/kg/min Ang II (Calbiochem, CA, USA) for 28 days. Ang II was infused using Alzet osmotic pumps (DURECT, Cupertino, Calif, Model 2004). Mice were anesthetized with Isoflurane, delivered using a calibrated vaporizer equipped with an induction chamber and a nose cone, and administered at 2-5% in O_2_ (2 L/min). It was then decreased to 2% to maintain anesthesia. Anesthesia was monitored by pinching the toe. Pumps were implanted subcutaneously in the back in the prone position through a small incision that was closed with suture.

**Figure 1:**
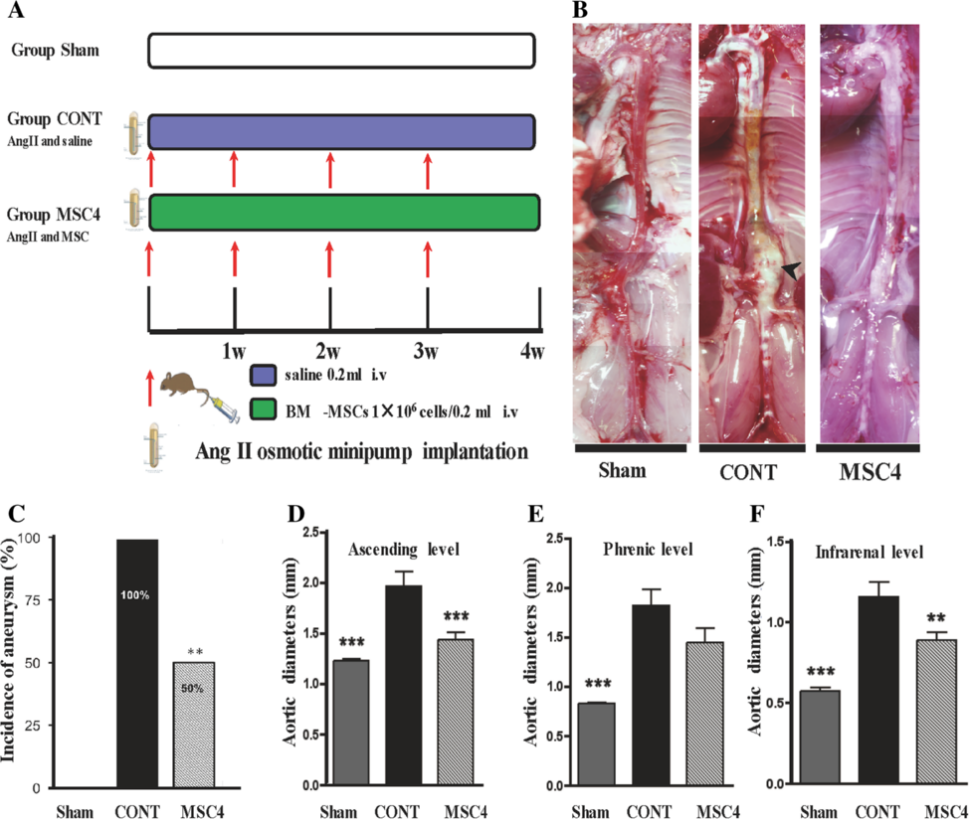
**Multiple intravenous administrations of BM-MSCs inhibited AngII-induced aortic aneurysm (AA) formation in apoE**^**−/− **^**mice. A)** Schematic diagram of study design. **B)** Representative macroscopic images of aorta from group Sham, CONT, and MSC4. Arrowheads indicate typical AAs induced by Ang II in apoE^−/−^ mice. **C)** Incidence of Ang II-induced AA was significantly decreased in group MSC4 compared with group CONT. There was no AA formation in group Sham. **D-F)** Aortic outer diameters measured at ascending, phrenic, and infrarenal levels in apoE^−/−^ mice. Data are presented as means ± SEM (*n* =10-12) **P<0.01, ***P<0.001 vs. group CONT, assessed by chi-square test (for AA incidence) and one-way ANOVA (for aortic diameter).

The apoE^−/−^ male mice were divided randomly into three groups: (1) a sham group (group Sham, *n*=10), mice without Ang II infusion or BM-MSCs administration, (2) a control group, mice with Ang II infusion and saline injection (group CONT, *n*=10), (3) and a MSCs treatment group, mice with Ang II infusion and multiple BM-MSCs intravenous administration (group MSC4, *n*=12). Because the effects of a single intravenous administration of BM-MSCs (group MSC1, n=10) were modest in our preliminary experiments (Additional files 2, 3, 4, 5), we established multiple BM-MSCs administration with the same dosage of cells (1×10^6^ MSCs in 0.2 ml saline), or 0.2 ml saline was injected weekly into apoE^−/−^ mice via tail vein at the time of Alzet osmotic pump implantation. Mice were intravenously administered BM-MSCs or saline four times totally (Figure 1A)*.*

To identify BM-MSCs in the aorta, suspended BM-MSCs on the day of transplantation were labeled with fluorescent dyes using a PKH26 red fluorescent cell linker kit (Sigma-Aldrich, St. Louis, MO), as reported previously [19].

### Aortic diameter measurement and specimen preparation

Twenty-eight days after pump implantation, all animals were euthanized by pentobarbital overdose. The aorta from ascending to the iliac bifurcation was dissected free from the surrounding connective tissue. The outer aortic diameters at the level of the ascending aorta, the abdominal aorta of the phrenic neck, and the abdominal aorta at the infrarenal proximal neck site were obtained with a calibrated ocular grid, as described previously [20]. Images of aorta were recorded with a digital camera and analyzed with ImageJ software (NIH).

A commonly used clinical standard to diagnose abdominal aortic aneurysm is an increase in aortic diameter of ≈50% [21]. The average diameter of normal suprarenal aorta in naïve control mice is ≈0.8 ± 0.01 mm. We therefore set a threshold of 1.25 mm as evidence of the incidence of aneurysm formation.

The aorta from the ascending aorta to the iliac bifurcation was then dissected away, and a 2-mm length of suprarenal aorta was cut for histologic examination. The remaining aorta was bisected longitudinally for elastin quantification, zymography, and enzyme-linked immunosorbent assay (ELISA).

### Measurement of elastin content in aortic tissue

Elastin was isolated as previously described [22]. The elastin amounts of aortas from apoE^−/−^ mice were quantified using a Fastin elastin assay kit (Biocolor, County Antrim, UK) according to the manufacturer’s protocol. Briefly, the dissected aortic tissues were weighed and cut into pieces with fine scissors. To convert insoluble elastin to water soluble alpha-elastin, approximately 1 mg (dry weight) of samples were placed with 300 μL of 0.25 M oxalic acid into a metal heating block with the thermostat set at 100°C for 60 min. The samples were then centrifuged at 3,000 × g for 10 min and 250 μL of the supernatant was retained in a new container with 250 μL of elastin precipitating reagent. The tubes were centrifuged at 10,000 × g for 10 min and the supernatants discarded, followed by adding 1.0 mL of the dye reagent, and were placed in a mechanical shaker at room temperature for 90 min. Subsequently, the tubes were centrifuged at 10,000 × g for 10 min and the supernatants discarded. Two-hundred fifty μL of dye dissociation reagent was added to the elastin-bound dye pellet to release the bound dye into solution. Aliquots of each sample (250 μL) were transferred to the wells of a 96-well plate and the optical density was measured at 513 nm. Elastin values were standardized to the corresponding dry-weight.

### Gelatin zymography

Gelatin zymography was performed to evaluate MMP-2 and MMP-9 enzymatic activities in aortic tissues as described previously [23]. Briefly, the aortic tissues *in vivo* were homogenized with a protein extraction buffer containing 20 mM Tris–HCl (pH 7.5) and 0.01% Brij-35 (MP Biomedicals, CA, USA). The protein concentration in each lysate was measured using a BCA assay kit (PIERCE, Rockford, IL, USA). Forty μL of an equal concentration of protein (0.2 mg/mL) was applied to the samples for electrophoresis, and was detected using a Gelatin Zymography electrophoresis kit (Life Kenkyusho, Yamagata, Japan) according to the manufacturer’s protocol. To quantitatively assess the intensity of bands for MMPs quantitatively, densitometric analysis was performed using Digi Print Doc (Bio tools, Gunma, Japan) with imaging Quant TL (GE Healthcare, Bucks, UK).

### Histology and immunofluorescence staining

Suprarenal aortas from each group were embedded in OCT compound (Tissue-Tek, Miles Inc., IN, USA). Specimens were cut into 5-mm cross-sections, and stained with Elastica Van Gieson (EVG) for elastin fibers. For identifying macrophages in the aortic wall *in vivo*, rat anti-mouse polyclonal antibody against F4/80 (1:100, AbDSerotec, Oxford, UK) was used, and the secondary antibody Alexa, Fluor 488 conjugated anti-rat IgG (H+L) (1:1,000, Cell Signaling Technology, Boston, MA, USA) was used for detection. Nuclei were stained with 40, 6-diamidino-2-phenyindole, DAPI (Vector Laboratories, Burlingame, CA, USA). Slides were examined with Olympus IX51 microscope and images captured using DP2-BSW software (Olympus, Tokyo, Japan). Quantification of F4/80 staining area was performed by two blinded investigators in 10 randomly selected areas and subsequently averaged using the Image-Pro Plus version 4.1 software (Media Cybernetics, Inc., MD).

### Quantitative protein expression of aortic tissue

The quantitative expression of protein including MMP inhibitors (TIMP-1 and −2), chemokine (MCP-1), cytokines (IL-1ß, IL-6, and TNF-α), and growth factors (IGF-1 and TGF-ß1) was determined by enzyme-linked immunosorbent assay (ELISA) as described previously [23]. Aortic tissues were homogenized in protein extraction buffer (CytoBusterTM; Novagen, Merck KGaA, Darmstadt, Germany) with 20 mM EDTA (Dojindo, Kumamoto, Japan) and 1 mM PMSF (Thermo, Fisher Scientific, MA, USA). The protein concentration for the lysate was measured using a BCA assay kit (Pierce). An equal concentration of total protein was applied to samples for each plate and detected according to the manufacturer’s protocol of each ELISA kit (IGF-1; Mediagnost, Reutlingen, Germany, TGF-ß1, TIMP-1; R&D Systems, Minneapolis, MN, USA, TIMP-2; RayBiotech, Norcross, GA, USA, IL-1ß, IL-6, TNF-α and MCP-1; Bender MedSystems, Vienna, Austria).

### Statistical analyses

Data analyses were performed with GraphPad Prism for Mac (Version 4, San Diego, CA, USA). Results were expressed as mean ± SEM. Statistic comparison for the incidence of AA was performed by chi-square test. Multiple comparisons of mean values were performed by a one-way factorial analysis of variance (ANOVA), and unpaired *t*-tests. Statistical significance was defined as *P*<0.05.

## Results

### BM-MSCs inhibited the development of AA in Ang II-infused apoE^−/−^ mice

To determine the effects of intravenous administration of BM-MSCs on Ang II-induced AA formation, AngII-infused apoE^−/−^ mice received multiple intravenous administrations of BM-MSCs or saline via tail vein. Figure 1B shows representative images of aortas from each group. The incidence of the development of AA in group CONT was 100%, which was significantly decreased to 50% in group MSC4 (Figure 1C). Then, the aortic outer diameter at the ascending, phrenic, and infrarenal level in each group was measured. At those three levels, group CONT mice showed a significantly larger aortic diameter compared with group Sham. Treatment with BM-MSCs resulted in significantly decreased aortic diameters compared with group CONT at the levels of ascending and infrarenal, but not at the phrenic level (Figure 1D**-**F). These results indicated that AngII infusion for 28 days in apoE^−/−^ mice induced AAs and that this process could be effectively inhibited by multiple intravenous administrations of BM-MSCs.

### BM-MSCs suppressed aortic elastin degradation in AngII-infused apoE^−/−^ mice

**Figure 2:**
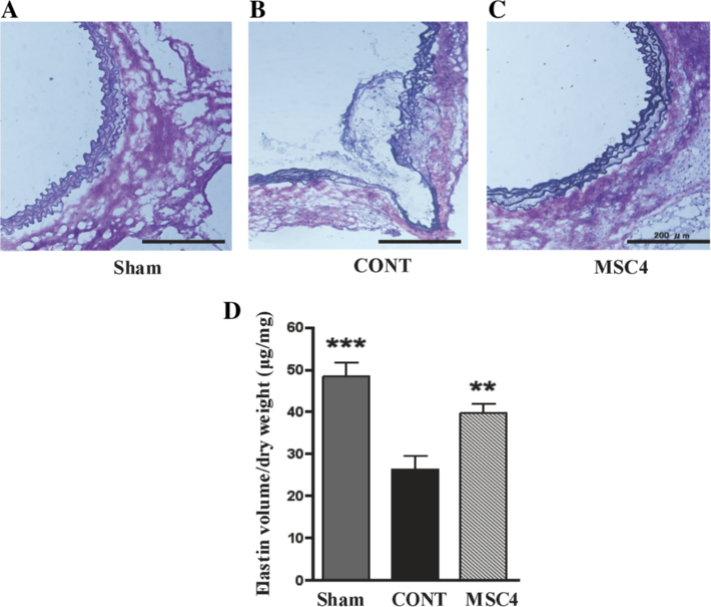
**Multiple intravenous administrations of BM-MSCs attenuated aortic elastin degradation in apoE**^**−/− **^**mice. A)** EVG staining shows normal wavy elastic lamina structure in group Sham, and **B)** disruption of elastic lamina and aneurysm formation in group CONT. **C)** Administration of BM-MSCs maintained wavy structure of the elastic lamellae. Scale bar = 200 μm. **D)** Measurement of elastin volume of aortic tissues showed a significant decrease in group CONT compared with group Sham, but preservation in group MSC4. Data are presented as means ± SEM (*n* =10-12) **P<0.01, ***P<0.001 vs. group CONT, assessed by one-way ANOVA.

EVG-stained sections from suprarenal aortas of group Sham showed normal wavy elastic lamina structure in the aortas, but sections from group CONT showed disruption of elastic fibers and aneurysm formation. Intravenous administration of MSCs partially maintained the wavy structure of the elastic lamellae (Figure 2A**-**C). In addition, elastin volume of the aortic tissues was measured. The elastin content of the aorta in group MSC4 was significantly increased compared with group CONT, and showed no significant difference compared with group Sham (group MSC4 vs. group CONT, 39.69 ± 7.65 vs 24.80 ± 2.78, μg/mg, *P* < 0.01; group MSC4 vs. group Sham, 39.69 ± 2.21 vs. 48.35 ± 3.28, μg/mg, *P* > 0.05; Figure 2D).

### BM-MSCs decreased inflammatory response in AngII-infused apoE^−/−^ mice

**Figure 3:**
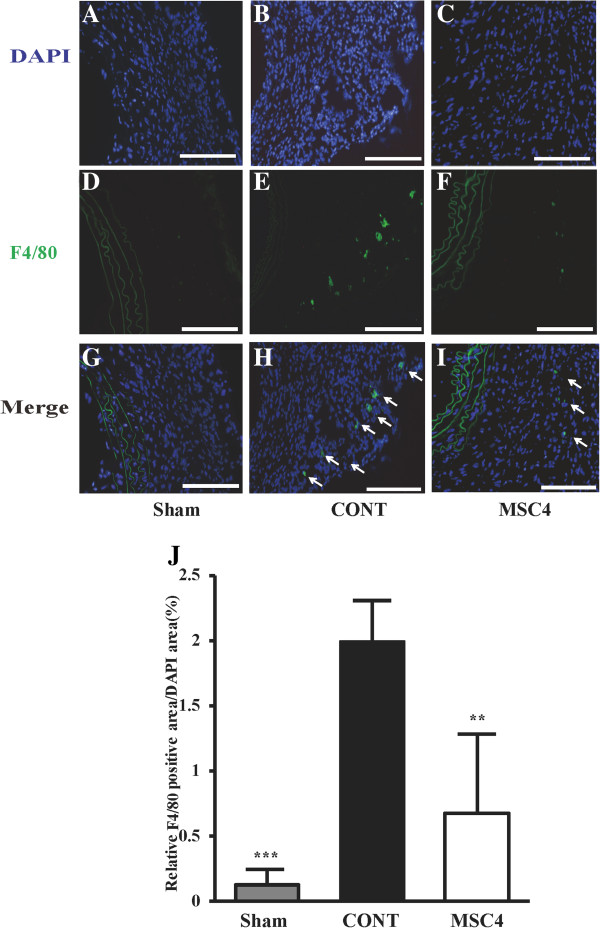
**Multiple intravenous administrations of BM-MSCs suppressed macrophage infiltration in aortic tissues. A-I)** Representative F4/80 immunofluorescence stained sections of suprarenal aortas from Sham, CONT, and MSC4 groups. Arrowheads indicated F4/80 macrophages. Scale bars=100 μm. **J)** Quantitation of F4/80- positive macrophages area as a ratio of the DAPI staining area. Data are presented as means ± SEM (*n* =10-12) **P<0.01, ***P<0.001 vs. group CONT, assessed by one-way ANOVA.

Immunofluorescence staining demonstrated that F4/80-positive macrophages were detected abundantly in the adventitia and media of the suprarenal aortic walls of group CONT. The macrophages infiltration was ameliorated by BM-MSCs administration (Figure 3 A**-**E). Quantitation of the area of F4/80 staining as a ratio of the DAPI staining area was shown in Figure 3 J.

### BM-MSCs suppressed MMP-2 and MMP-9 activity in AngII-infused apoE^−/−^ mice

**Figure 4:**
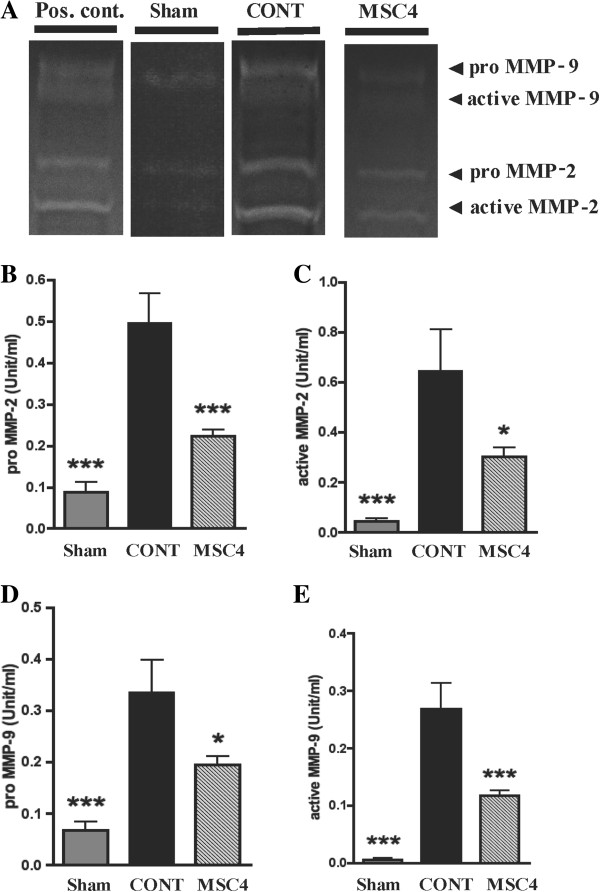
**Gelatin zymography of MMPs activities in aortic tissues. A)** Gelatin zymography of 4 representative specimens from positive control (from kit), and Sham, CONT, and MSC4 groups. **B-E)** Zymographic band densities from all tissue samples were quantified by densitometry. Enzyme activities of (pro- and active-) MMP-2 and MMP-9 were significantly decreased in group MSC4 compared with group CONT. The enzyme activities of MMPs are expressed as a mean ±SEM (*n* =10-12). *P < 0.05. ***P <0.001 vs. group CONT, assessed by one-way ANOVA. MMPs, matrix metalloproteinases.

Gelatin zymography was conducted to evaluate MMP-2 and -9 enzymatic activities in the aortic tissues. A representative zymogram was shown in Figure 4A. Quantitation of the band intensity shows that (pro- and active-) MMP-2 and (pro- and active-) MMP-9 activities were significantly decreased in group MSC4 compared with group CONT (Figure 4B**-**E).

### BM-MSCs regulated aortic cytokines, chemokine, and growth factors expression

**Figure 5:**
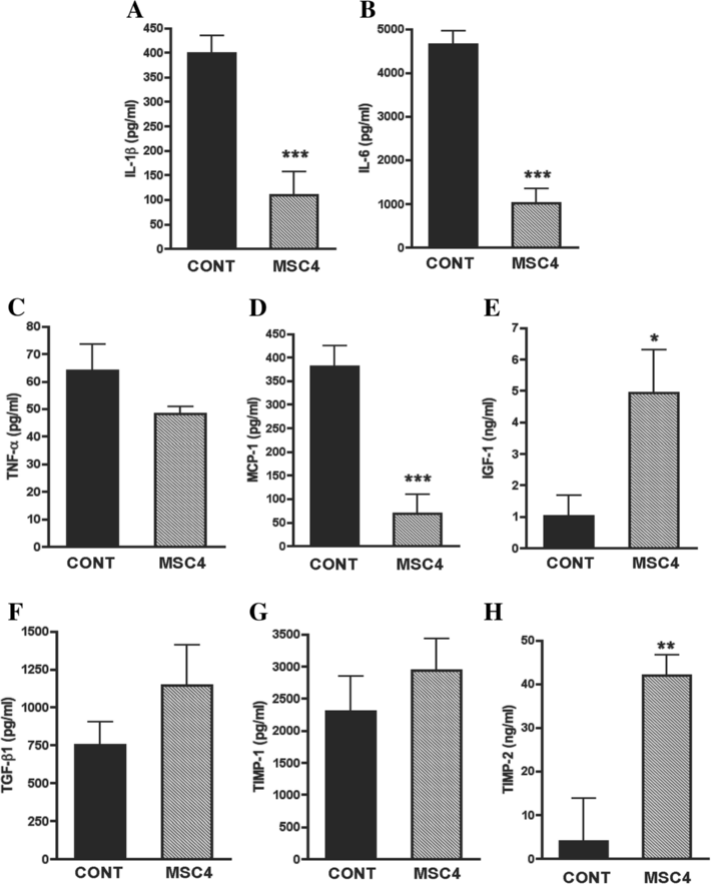
**ELISA analyses of inflammatory cytokines (IL-1β, IL-6, and TNF-α), chemokine (MCP-1), tissue inhibitor of metalloproteinases (TIMP-1 and TIMP-2), and growth factors (IGF-1 and TGF-β1) in aortic tissues 4 weeks after operation.** Intravenously administered BM-MSCs reduced IL-1β **(A)**, IL-6 **(B)**, and MCP-1 **(D)** but did not significantly alter expression of TNF-α **(C)**. BM-MSCs also increased expressions of IGF-1 **(E)**, and TIMP-2 **(H)** but did not alter expressions of TGF-β1 **(F)** and TIMP-1 **(G)**. All data are presented as mean ± SEM (*n* =10-12). *P < 0.05, **P < 0.01, and ***P < 0.001 vs. group CONT, assessed by unpaired *t*-test. IL, interleukin; TNF, tumor necrosis factor; MCP, monocyte chemotactant protein; TGF-β, transforming growth factor β; IGF-1, insulin-like growth factor; TIMP, tissue inhibitor of metalloproteinase.

Protein expression including MMP inhibitors (TIMP-1 and TIMP-2), chemokine (MCP-1), proinflammatory cytokines (IL-1ß, IL-6, and TNF-α), and growth factors (IGF-1 and TGF-ß1) was evaluated by ELISA. There were no statistical differences in TNF-α expression between group CONT and group MSC4, while expressions of IL-1β, IL-6, and MCP-1 that promote inflammatory reaction were significantly decreased in group MSC4 compared with group CONT (Figure 5A**-**D). At the same time, the expression of IGF-1 and TIMP-2 which promote elastin synthesis were significantly increased in group MSC4 compared with group CONT, though expression of TGF-β1 and TIMP-1 showed no significant difference (Figure 5E**-**H).

### Cell tracking of transplanted BM-MSCs

**Figure 6:**
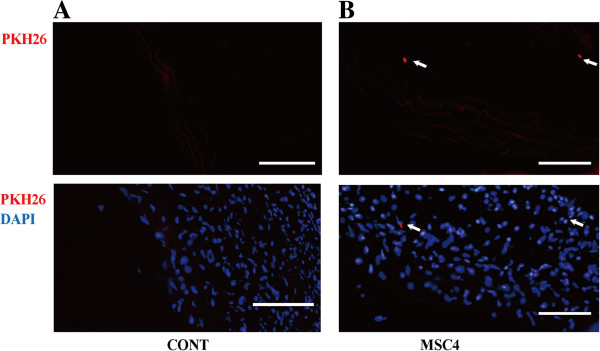
**Tracking of transplanted PKH26 labeled BM-MSCs in the aortic walls. A)** Representative aortic sections from group CONT. **B)** Representative aortic sections from group MSC4. Four weeks after operation, PKH26-positive BM-MSCs were mainly observed in the adventitia of aorta in group MSC4. Arrows point to sites of BM-MSC. At least three random aortic cross sections per mouse were chosen for evaluation. PKH26 (red), DAPI (blue), Scale bars = 100 μm.

We investigated whether intravenous injected BM-MSCs mobilized into the aortic walls. BM-MSCs were labeled with fluorescent PKH26 and then intravenously injected into the apoE^−/−^ mice via tail vein. PKH26 labeled BM-MSCs were detected in the media and adventitia of aortas four weeks after operation (Figure 6).

## Discussion

In the present study, we demonstrated that intravenously-administered BM-MSCs were capable of engrafting into the aortic wall in Ang II-infused apoE^−/−^ mice, and that multiple intravenous administrations of BM-MSCs could effectively: (1) reduce the incidence of AA, (2) suppress both inflammatory reaction and elastin degradation in aortic tissues, and (3) inhibit MMP-2 and MMP-9 activities in the aortic tissues. Here, for the first time, we showed that multiple intravenous administrations of BM-MSCs were effective for prevention of Ang II-induced AA in apoE^−/−^ mice.

Ang II-induced AA in apoE^−/−^ mice provides a model that resembles human AA. Ang II promotes vascular inflammation characterized by macrophage infiltration in the vascular wall [24,25]. Macrophages are a major source of proteolytic enzymes like MMPs, which can degrade the extracellular matrix and impair the structural integrity of the vascular wall, leading to AA formation [25]. On the other hand, it has been reported that activated macrophages produce a number of proapoptotic mediators, which result in apoptosis of SMCs [26]. Thus, vascular inflammation, which triggers both vascular wall proteolysis and SMCs apoptosis, could result in an imbalance between extracellular matrix degradation and synthesis, favoring tissue destruction and AA formation. Accumulating evidence indicates that anti-inflammation is a promising therapeutic strategy for AA [4]. MSCs reportedly have anti-inflammation activities [27]. This anti-inflammatory effect of MSCs could inhibit both the proteolytic and proapoptotic pathways, thus restoring the balance between matrix synthesis and degradation, thereby improving tissue repair.

We previously reported that intra-abdominal implantation of BM-MSCs sheets by laparotomy inhibited the development of Ang II-induced aortic aneurysm in apoE^−/−^ mice by anti-inflammation and elastin preservation [16]. However, BM-MSCs implantation by laparotomy is an invasive approach; an alternative delivery approach is needed. Numerous experimental studies have shown that intravenous delivery of MSCs is an effective approach for treatment of a variety of diseases such as myocardial infarction [28], stroke [29], lung injury [30], and diabetes mellitus [31]. Moreover, it is considered a suitable route for translation into clinical application, due to its low invasiveness. In the present study, we investigated the effects of intravenous administrations of BM-MSCs on AngII- induced AA formation in apoE^−/−^ mice. In our preliminary experiments we found that the effects of single intravenous administration of BM-MSCs were modest (data was shown in additional figures), then we focused on multiple intravenous administrations of BM-MSCs. Our results demonstrated that multiple intravenous administrations of BM-MSCs significantly reduced the incidence of Ang II-induced AA in apoE^−/−^ mice.

Consisted with our previous study, we found that multiple intravenous administrated BM-MSCs suppressed infiltration of macrophages and activities of MMPs in the aortic walls. BM-MSCs also markedly decreased proinflammatory cytokines secretions including IL-1β, IL-6, and MCP-1, while increasing growth factor expressions like IGF-1 and TIMP-2, which promote elastin synthesis. These results suggested that the effects of multiple intravenous administrated BM-MSCs on Ang II-induced AA in apoE^−/−^ mice may be associated with anti-inflammatory actions.

It should be noted that in this study multiple intravenous administrations of BM-MSCs effectively inhibited development of AngII-induced AA in apoE^−/−^ mice, while single intravenous administration of BM-MSCs showed modest effects. We presumed that the timing and cell dose of BM-MSCs might account for the different results. BM-MSCs were shown to be able to migrate into sites of inflammation or injury when transplanted locally or systemically [32], which was believed to be mediated by chemotactic proteins such as MCP-1, and IL-8 secreted from injured or inflammatory tissues [33]. The timing of single BM-MSCs injection in this study was just after pump implantation, and at that time aortic inflammation did not occur. On the other hand, we intravenously administrated 4×10^6^ BM-MSCs for each mouse in multiple injection group while 1×10^6^ in single injection group. The different cell dose may affect the strength of anti-inflammatory effects of BM-MSCs. The appropriate timing and cell dose for intravenous administrations of BM-MSCs treatment of AA remain unclear. Further studies are currently under investigation by our group.

There are several limitations to the present study. First, the BM-MSCs population used in this experiment may be mixed, rather than limited to BM-MSCs, although cell surface markers of cultured cells were consistent with those previously reported [18]. Second, although we observed the effects of multiple intravenous administrations of BM-MSCs 4 weeks after operation, the long-term effects remain unclear, and further experiment is needed. Third, we used a model of Ang II-induced AA in apoE^−/−^ mice, further experiments using other AA models are needed to confirm the effects of multiple intravenous administrations of BM-MSCs.

## Conclusions

Multiple intravenous administrations of BM-MSCs were effective to suppress inflammatory reactions in Ang II-infused apoE^−/−^ mice, and inhibit the development of AAs. It may therefore serve as a new therapeutic strategy for patients with AA.

## Competing interests

The authors declare that they have no competing interests.

## Authors’ contributions

XF, AY, and YN designed the experiment, interpreted results. XF drafted manuscript. XF and AY performed experiments. YN, HO, YU, and AU made critical revision to manuscript. All authors have read and approved the final manuscript.

## Supplementary Material

Additional file 1: Figure 1 Characterization of BM-MSCs. A) Morphology of BM-MSC. Scale bar=100 μm. B-D) Multipotency of BM-MSCs. BM-MSCs differentiated into osteocytes (B), adipocytes (C), and chondrocytes (D). Scale bars=100 μm. E) Flow cytometric analysis of BM-MSCs. Click here for file

Additional file 2: Figure 2 Single intravenous administration of BM-MSCs did not inhibit Ang II-induced aortic aneurysm formation in apoE^−/−^ mice. A) Incidence of Aortic aneurysm. B-D) Aortic outer diameters measured at ascending, phrenic, and infrarenal levels in apoE^−/−^ mice. Data are presented as means ± SEM (*n* =10-12) **P<0.01, ***P<0.001 vs. group CONT, assessed by chi-square test (for AA incidence) and one-way ANOVA (for aortic diameter). Click here for file

Additional file 3: Figure 3 Single intravenous administration of BM-MSCs did not attenuate aortic elastin degradation in apoE^−/−^ mice. A) EVG staining of suprarenal aortas and B) elastin volume of aortic tissues. Data are presented as means ± SEM (*n* =10-12) ***P<0.001 vs. group CONT, assessed by one-way ANOVA. Click here for file

Additional file 4: Figure 4 Single intravenous administration of BM-MSCs did not suppress macrophages infiltration in aortic tissues. Representative F4/80 immunohistochemical (A- I) stained sections of suprarenal aortas from Groups Sham, CONT, and MSC1. Scale bars=100 μm. *J*) Quantitation of F4/80-positive macrophages. Data are presented as means ± SEM (*n* =10-12) **P<0.01, ***P<0.001 vs. group CONT, assessed by one-way ANOVA. Click here for file

Additional file 5: Figure 5 Gelatin zymography of MMPs activities in aortic tissues. (*A*-*D*) Zymographic band densities were quantified by densitometry. Enzyme activities (pro- and active-) of MMP-2 and MMP-9 are expressed as a mean ±SEM (*n* =10-12). ***P < 0.001 vs. group CONT, assessed by one-way ANOVA. MMPs, matrix metalloproteinases. Click here for file
