# Supplementary figures and images for: Intravenous administration of mesenchymal stem cells prevents angiotensin II-induced aortic aneurysm formation in apolipoprotein E-deficient mouse
Source: J Transl Med. 2013 Jul 22;11:175. doi: 10.1186/1479-5876-11-175 (PMC3726376; doi:10.1186/1479-5876-11-175)

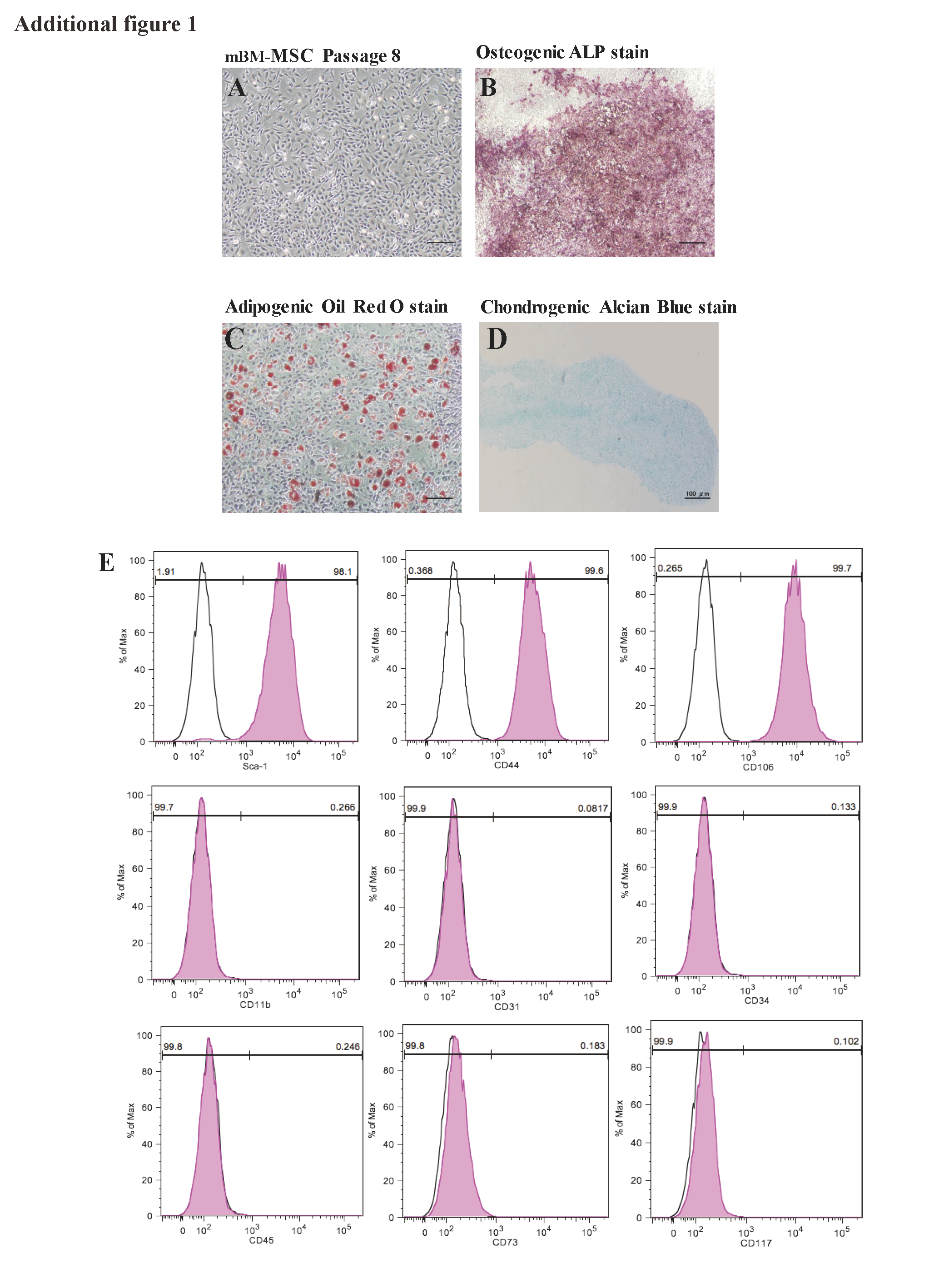

Supplement: Additional file 1: Figure 1 — Characterization of BM-MSCs. A) Morphology of BM-MSC. Scale bar=100 μm. B-D) Multipotency of BM-MSCs. BM-MSCs differentiated into osteocytes (B), adipocytes (C), and chondrocytes (D). Scale bars=100 μm. E) Flow cytometric analysis of BM-MSCs. [file 1479-5876-11-175-S1.tiff]

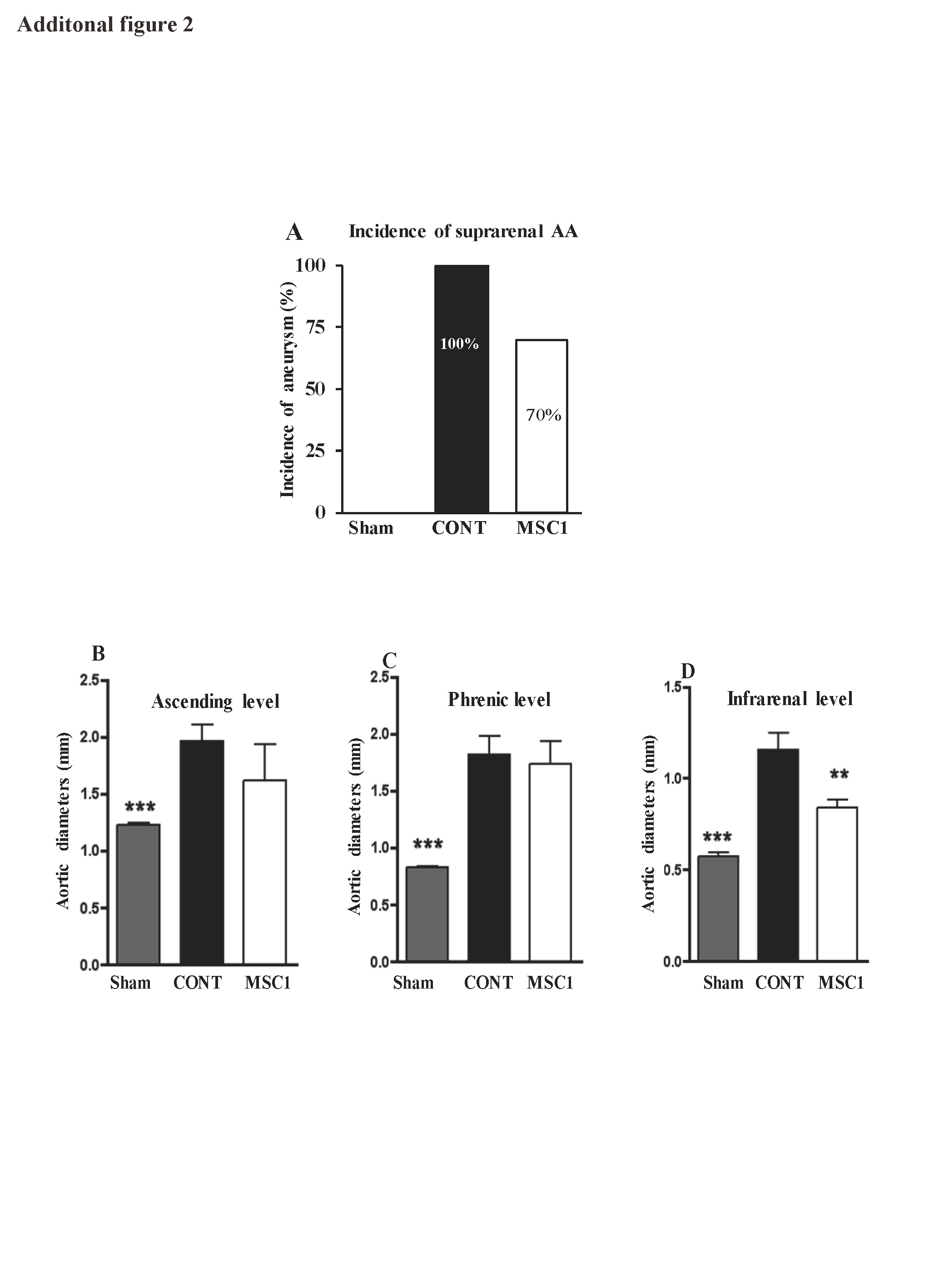

Supplement: Additional file 2: Figure 2 — Single intravenous administration of BM-MSCs did not inhibit Ang II-induced aortic aneurysm formation in apoE−/− mice. A) Incidence of Aortic aneurysm. B-D) Aortic outer diameters measured at ascending, phrenic, and infrarenal levels in apoE−/− mice. Data are presented as means ± SEM (n =10-12) **P<0.01, ***P<0.001 vs. group CONT, assessed by chi-square test (for AA incidence) and one-way ANOVA (for aortic diameter). [file 1479-5876-11-175-S2.tiff]

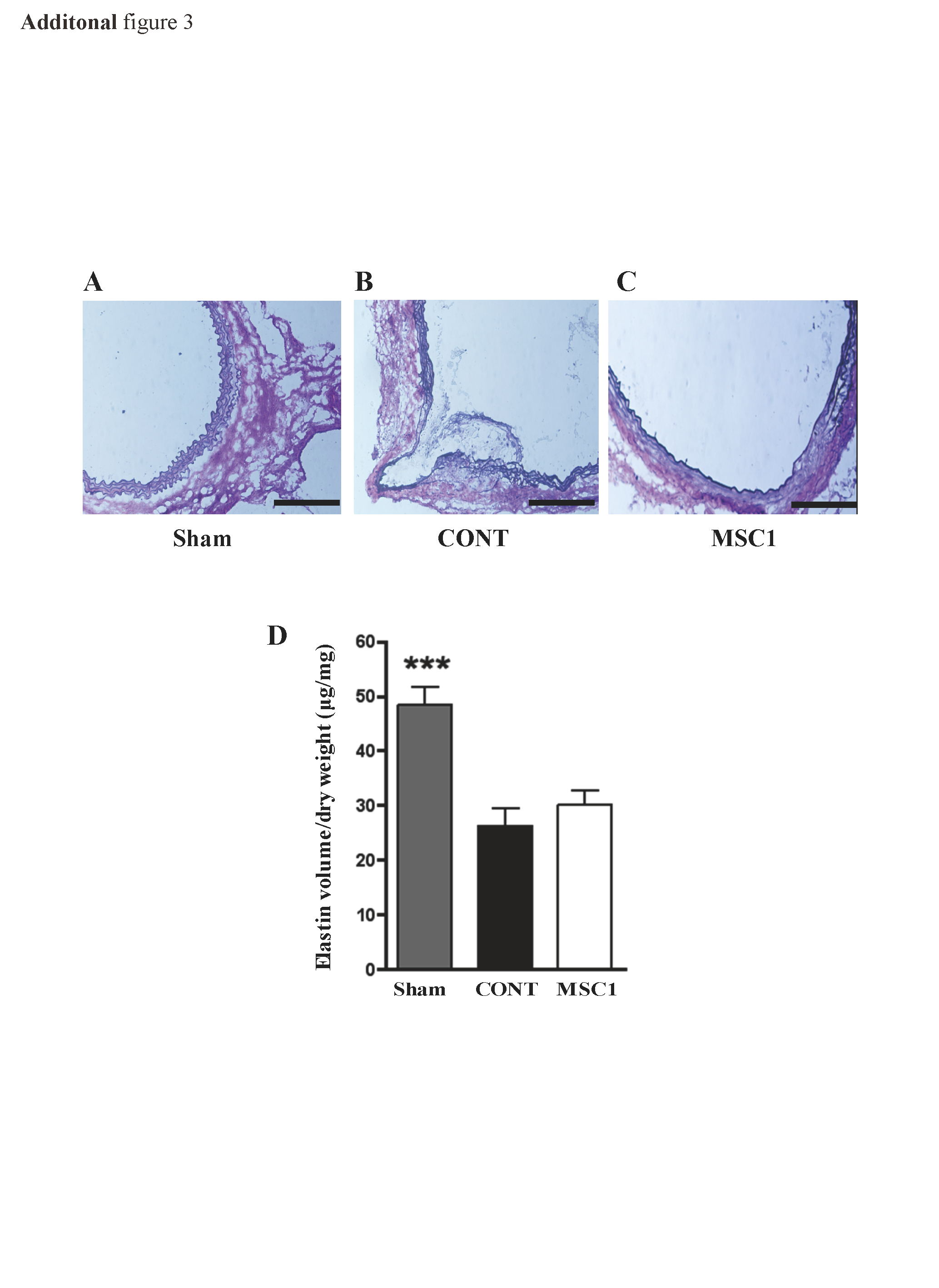

Supplement: Additional file 3: Figure 3 — Single intravenous administration of BM-MSCs did not attenuate aortic elastin degradation in apoE−/− mice. A) EVG staining of suprarenal aortas and B) elastin volume of aortic tissues. Data are presented as means ± SEM (n =10-12) ***P<0.001 vs. group CONT, assessed by one-way ANOVA. [file 1479-5876-11-175-S3.tiff]

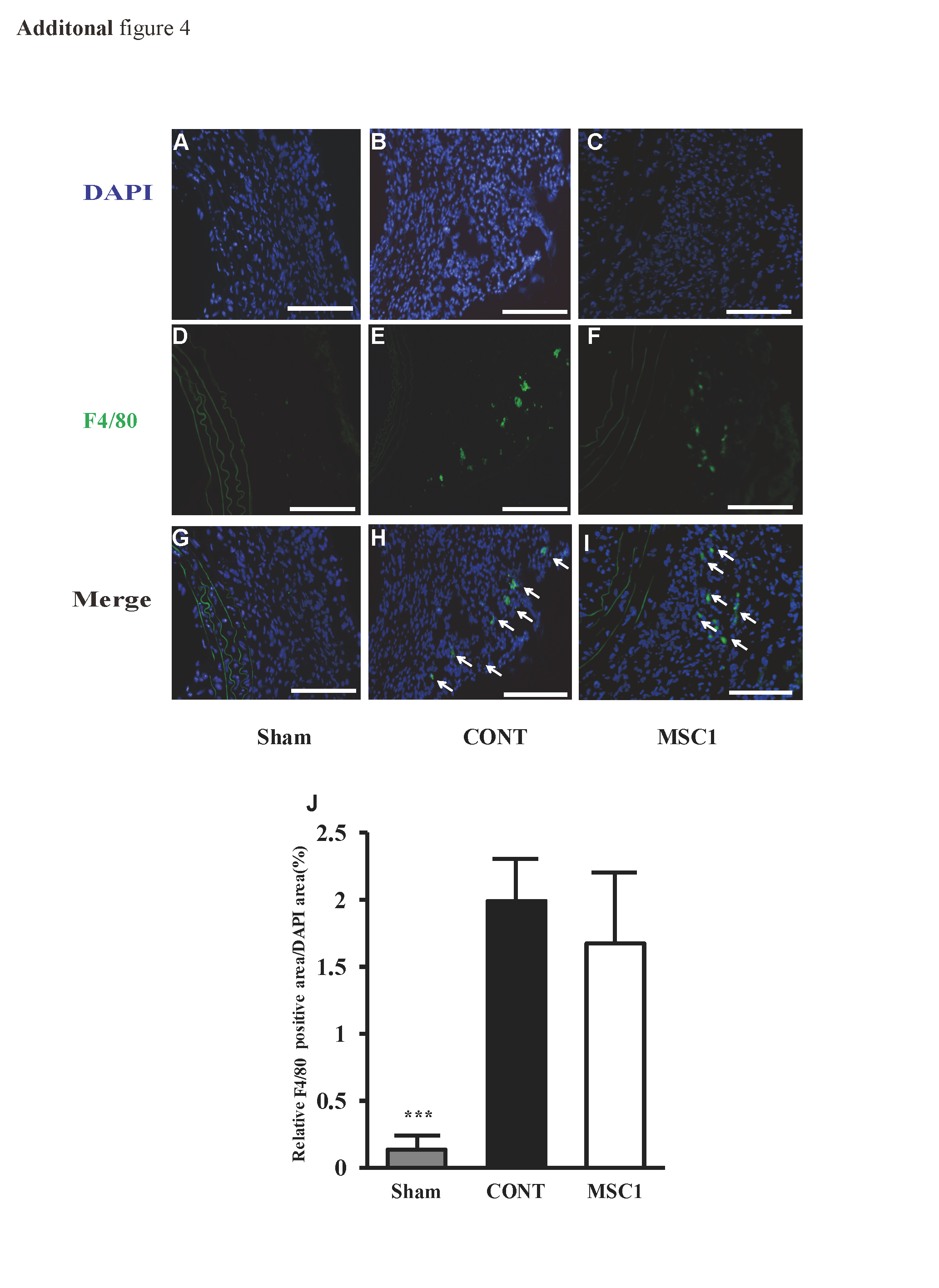

Supplement: Additional file 4: Figure 4 — Single intravenous administration of BM-MSCs did not suppress macrophages infiltration in aortic tissues. Representative F4/80 immunohistochemical (A- I) stained sections of suprarenal aortas from Groups Sham, CONT, and MSC1. Scale bars=100 μm. J) Quantitation of F4/80-positive macrophages. Data are presented as means ± SEM (n =10-12) **P<0.01, ***P<0.001 vs. group CONT, assessed by one-way ANOVA. [file 1479-5876-11-175-S4.tiff]

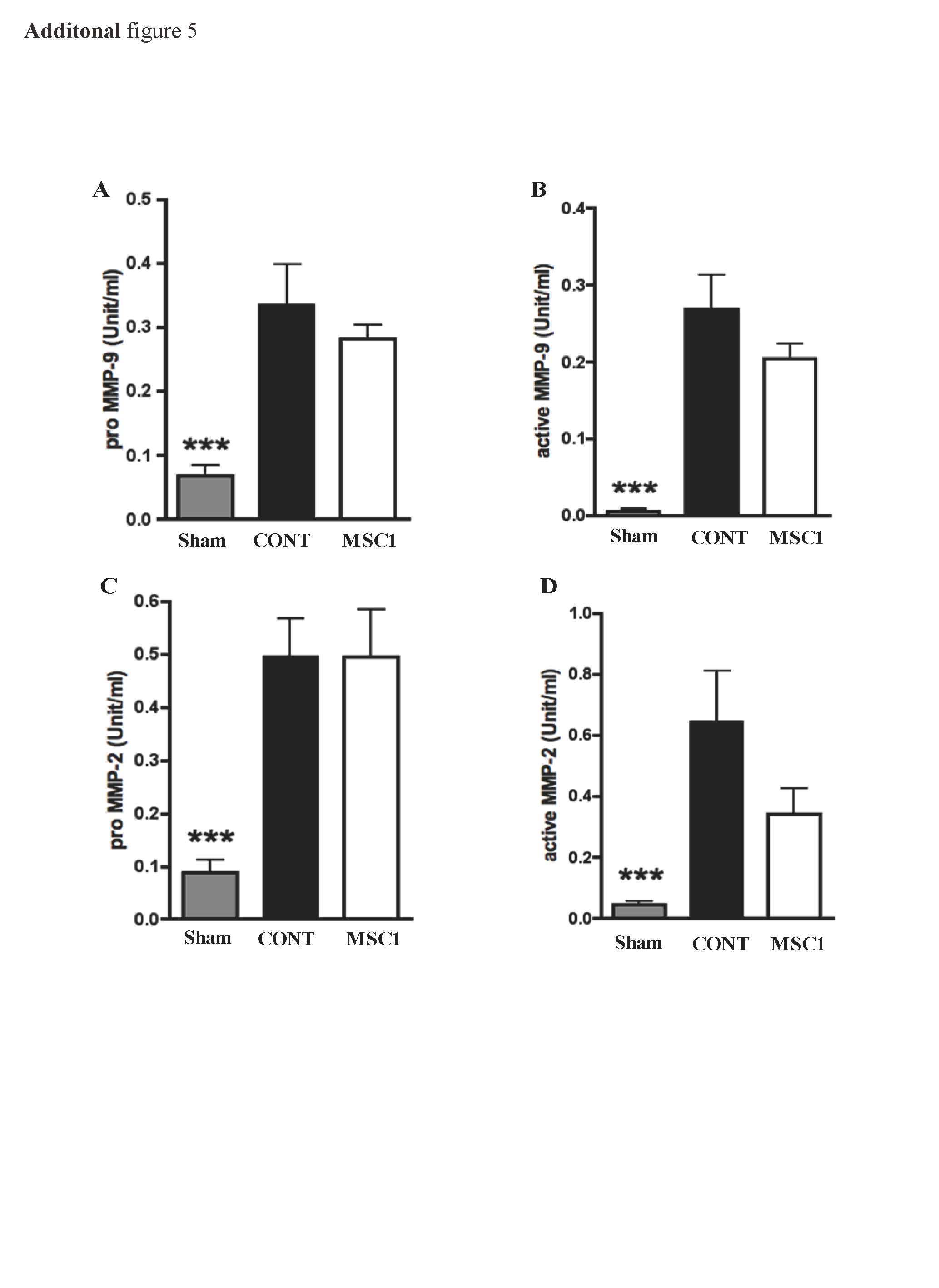

Supplement: Additional file 5: Figure 5 — Gelatin zymography of MMPs activities in aortic tissues. (A-D) Zymographic band densities were quantified by densitometry. Enzyme activities (pro- and active-) of MMP-2 and MMP-9 are expressed as a mean ±SEM (n =10-12). ***P < 0.001 vs. group CONT, assessed by one-way ANOVA. MMPs, matrix metalloproteinases. [file 1479-5876-11-175-S5.tiff]
